# Inflammatory Biomarkers in the Short-Term Prognosis of Venous Thromboembolism: A Narrative Review

**DOI:** 10.3390/ijms22052627

**Published:** 2021-03-05

**Authors:** Francisco Galeano-Valle, Lucía Ordieres-Ortega, Crhistian Mario Oblitas, Jorge del-Toro-Cervera, Luis Alvarez-Sala-Walther, Pablo Demelo-Rodríguez

**Affiliations:** 1 Venous Thromboembolism Unit, Internal Medicine, Hospital General Universitario Gregorio Marañón, Calle Doctor Esquerdo, 46, 28007 Madrid, Spain; lucia.oomere@gmail.com (L.O.-O.); crhistian.cao@gmail.com (C.M.O.); jorgedeltoro@telefonica.net (J.d.-T.-C.); pbdemelo@hotmail.com (P.D.-R.); 2 School of Medicine, Universidad Complutense de Madrid, Plaza de Ramón y Cajal, s/n, 28040 Madrid, Spain; lalvarezsalaw@gmail.com; 3 Sanitary Research Institute Gregorio Marañón, Calle Doctor Esquerdo, 46, 28007 Madrid, Spain; 4 Internal Medicine, Hospital General Universitario Gregorio Marañón, Calle Doctor Esquerdo, 46, 28007 Madrid, Spain

**Keywords:** biomarkers, deep vein thrombosis, inflammation, pulmonary embolism, prognostic, reactive C protein, P-selectin, venous thromboembolism

## Abstract

The relationship between inflammation and venous thrombosis is not well understood. An inflammatory response may be both the cause and consequence of venous thromboembolism (VTE). In fact, several risk factors of VTE modulate thrombosis through inflammatory markers. Acute pulmonary embolism (PE) is burdened by a remarkable mortality rate, up to 34% in severely ill patients presenting with hemodynamic instability. Initial mortality risk stratification is based on hemodynamic instability. Patients with a situation of hemodynamic stability require immediate further risk assessment based on clinical, imaging, and circulating biomarkers, as well as the presence of comorbidities. Some inflammatory biomarkers have shown potential usefulness in the risk stratification of patients with VTE, especially acute PE. C-reactive protein on admission is associated with 30-day mortality and bleeding in VTE patients. P-selectin is associated with right ventricle dysfunction in PE patients and might be associated with VTE recurrences and the extension of thrombosis. Tissue factor microparticles are associated with VTE recurrence in cancer-associated thrombosis. Other inflammatory biomarkers present scarce evidence (inflammatory cytokines, erythrocyte sedimentation rate, fibrinogen, leukocyte count). In this manuscript, we will review the prognostic role of different inflammatory biomarkers available both for clinical practice and research in VTE patients.

## 1. Introduction

Deep vein thrombosis (DVT) and pulmonary embolism (PE) are the main manifestations of venous thromboembolism (VTE). DVT mostly occurs in the lower limbs, although the upper limbs, cerebral veins, and splanchnic territory may also be affected [1]. DVT and PE share common risk factors, and, in most cases, PE arises as a consequence of DVT [2]. PE is also the main cause of VTE-associated mortality and an important cause of in-hospital preventable mortality [3]. The estimated incidence of VTE in Europe is 1–1.8 per 1000 inhabitants [4].

Blood hypercoagulability, circulatory stasis, and endothelial disruption are considered the pillars of the pathophysiology of VTE. Certain provoking factors (either persistent, e.g., cancer, or transient, e.g., recent surgery) increase the risk of VTE [1,5]. A strong association between cancer and VTE has already been established. The relative risk of VTE is fairly higher in patients with active cancer, and 20% of all VTE occurs in patients with cancer [6]. Besides, other hereditary or acquired conditions (i.e., hereditary thrombophilias or antiphospholipid syndrome, respectively) might favor the development of VTE [7]. Nevertheless, more than a third of all VTE events are classified as idiopathic or nonprovoked [8].

Acute PE is burdened by a remarkable mortality rate, up to 34% in severely ill patients presenting with hemodynamic instability. However, when correctly diagnosed and promptly treated, acute PE is associated with a mortality rate close to 5% [9]. Initial mortality risk stratification is based on symptoms and signs of hemodynamic instability. Patients with a situation of hemodynamic stability require further immediate risk assessment based on clinical, imaging, and circulating biomarkers (mostly related to right ventricular (RV) function and myocardial injury), as well as the presence of comorbidities. Among circulating biomarkers, three groups of biomarkers can be considered: biomarkers of myocardial injury (troponins), biomarkers of right ventricular dysfunction (natriuretic peptides), and other biomarkers [10,11]. Among the currently available risk scores, the Pulmonary Embolism Severity Index (PESI) and its simplified version [12] and the European Society of Cardiology (ESC) guidelines recommend assessing troponins and natriuretic peptides levels in hemodynamically stable patients with PE to distinguish between intermediate/high-risk or intermediate/low-risk patients [11].

In this manuscript, the inflammatory mechanisms of VTE and inflammatory biomarkers useful for the short-term prognosis of VTE in clinical practice are reviewed.

## 2. Inflammation in VTE

**Figure 1:**
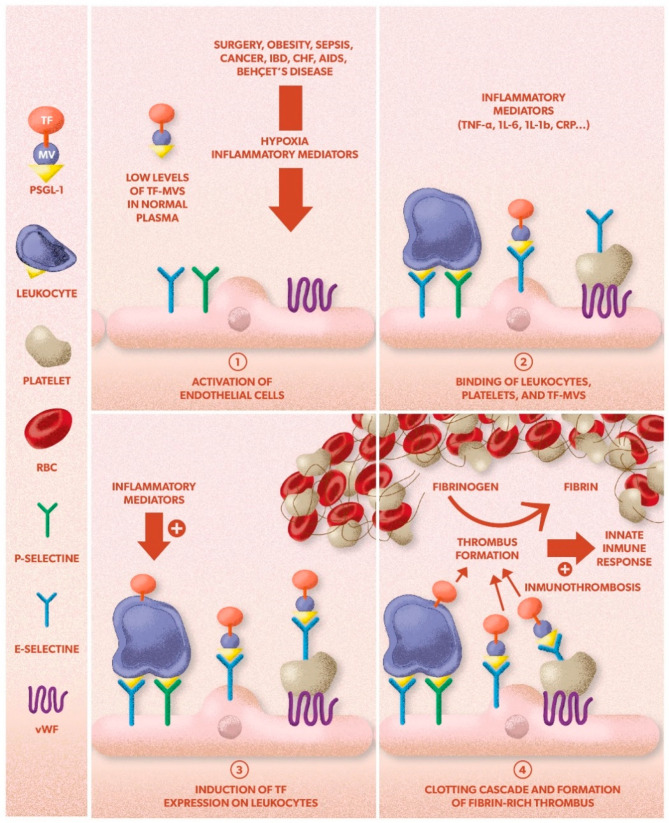
Proposed mechanisms for venous thrombosis. The formation of a venous thrombosis can be divided into distinct steps. ➀ The endothelium is activated by hypoxia and/or inflammatory mediators and expresses the adhesion proteins P-selectin, E-selectin, and vWF. ➁ Circulating leukocytes, platelets, and TF-MVs bind to the activated endothelium. ➂ The activation of endothelial cells, platelets, and leukocytes with the subsequent formation of MVs can trigger the coagulation system through the induction of TF. ➃ The local activation of the coagulation cascade overwhelms the protective anticoagulant pathways and triggers thrombosis. The fibrin-rich clot also contains platelets and red blood cells. AIDS: acquired immunodeficiency syndrome; CHF: congestive heart failure; CTEPH: chronic thromboembolic pulmonary hypertension; DVT: deep vein thrombosis; HR: hazard ratio; IBD: inflammatory bowel disease; MVs: microvesicles; PE: pulmonary embolism; PPV: predictive positive value; PTS: post-thrombotic syndrome; RV: right ventricle; TF: tissue factor; vWF: von Willebrand factor. Adapted from: Mackman N. New insights into the mechanisms of venous thrombosis. J Clin Invest. 2012;122(7):2331-6. Erratum in: J Clin Invest. 2012;122(9):3368. With permission from American Society for Clinical Investigation.

Little is known about the relationship between inflammation and venous thrombosis [13]. From a genetic point of view, certain gene variants of molecules related to inflammation (interleukin-10 (IL-10), interleukin-6 (IL-6), or interleukin-4 (IL-4)) have been linked to a higher risk of VTE, and some gene therapy studies performed in animal models have shown the role of inflammatory markers in the development of VTE [14] (Figure 1).

An inflammatory response may be both the cause and consequence of VTE [15]. In fact, several risk factors of VTE (surgery, obesity, sepsis, cancer) modulate thrombosis through inflammatory markers [15,16,17].

Immunothrombosis is a new concept, defined by Engelmann and Massberg [18] as the innate immune response induced by the formation of thrombus inside the blood vessels, particularly in microvessels. If uncontrolled, immunothrombosis could give an explanation to some cases of unprovoked VTE [17,19]. An inflammasome complex is a multimeric protein that activates as part of the innate response to pathogens, but its abnormal activation may induce cardiovascular disorders, including VTE [20].

Venous thrombosis is generally considered to be a pathological deviation of hemostasis and also involves coagulation, inflammation, and platelet activation [18,21,22]. Inflammation of the vessel wall may initiate thrombosis on an intact vein. The activation of endothelial cells, platelets, and leukocytes with subsequent formation of microparticles can trigger the coagulation system through the induction of tissue factor (TF) [15,16,17].

TF is synthesized by circulating monocytes and endothelial cells induced by inflammatory mediators, endotoxins [23], or the C-reactive protein [24]. Low levels of TF have been identified circulating in normal plasma, mainly associated with monocyte-derived microparticles [25], and may play a role in the thrombin generation in circulating blood [26].

During thrombus formation, platelets accumulate in the vessel wall, activate, and express P-selectin on the luminal surface of the thrombus [27]. P-selectin glycoprotein ligand 1 (PSGL-1) and P-selectin are vascular cell adhesion molecules that play a critical role in leukocyte trafficking and lymphocyte migration. P-selectin is expressed on activated platelets and binds to PSGL-1 on circulating leukocytes. Monocyte-derived microparticles expressing tissue factor and PSGL-1 accumulate on activated platelets expressing P-selectin, thus concentrating TF to a level that triggers the initiation of blood coagulation [25]. The bound microvesicles fuse with the platelets, transferring TF to the platelet surface [23]. In the absence of deep vessel injury and platelet deposition, López et al. [28] proposed a model where TF-bearing microvesicles interact with activated endothelium in a manner that resembles their interaction with activated platelets and therefore initiates venous thrombosis. A number of diseases associated with VTE are characterized by elevated levels of inflammatory mediators known to induce TF-bearing monocyte microvesicles, two examples being inflammatory bowel disease and chronic congestive heart failure [22]. Although the mechanisms responsible for the hypercoagulable state in these diseases are undoubtedly complex and multifactorial, the high levels of proinflammatory cytokines (e.g., TNF-a) characteristic of these conditions are likely to induce the generation of TF-bearing monocyte microvesicles. Similar prothrombotic mechanisms could operate in other inflammatory states characterized by both elevated cytokines known to induce TF expression (e.g., TNF-a, Il-6, Il-1b) and VTE development [22]. Examples of such states include Behçet disease [29], AIDS [30], cancer [31], systemic viral infections (notably CMV infection) [32], and surgery [33].

Inflammation can induce an incomplete resolution of the thrombus and a higher risk of VTE-associated complications, including post-thrombotic syndrome and chronic thromboembolic pulmonary hypertension. However, some inflammatory elements are essential for the proper resolution of VTE [15].

## 3. Inflammatory Biomarkers in VTE

In 2001, the Biomarkers Definitions Working Group defined “biomarker” as a characteristic that could be objectively measured and evaluated as an indicator of a normal biological process, a pathological process, or a pharmacological response to a therapeutic intervention [34]. They are, by definition, objective and quantifiable [35]. The ideal biomarker should be specific, sensitive, predictive, fast, economical, stable both in vitro and in vivo, noninvasive, and easily measurable and show sufficient preclinical and clinical relevance to modify the decisions relevant to the pathological process it is applied to [36,37].

Some inflammatory biomarkers have shown potential usefulness in the risk stratification of patients with VTE, especially acute PE. However, most of them are involved in different mechanisms (inflammation, coagulation, infection), which reduces their specificity [36]. In this manuscript, we will review the short-term usefulness of inflammatory biomarkers in VTE (Table 1 and Table 2, and Figure 1).

### 3.1. C-Reactive Protein (CRP)

CRP is a nonglycosylated protein, synthesized mainly in the hepatocyte [38,39]. It rises swiftly in response to a wide variety of infections and inflammatory diseases [40] and currently represents the paradigm of acute-phase reactants [39]. Its production is stimulated by cytokines, especially interleukin-1 (IL-1) and IL-6, and the tumoral necrosis factor (TNF) [39,40,41]. It is presently considered a modulator of innate immunity [42]. In healthy individuals, normal CRP levels are <1 mg/dL (normal mean levels increase with age); however, in pathological conditions, its levels increase in the first 6–8 h, reaching a peak of 35–40 mg/dL after approximately 48 h [40,43,44,45,46,47,48].

The association between the levels of CRP and the risk of developing VTE has been described in several prospective studies, including oncologic [49] and obese [50] populations [51] and a meta-analysis [43]. However, other studies have not confirmed this association [52]. Polymorphisms of the CRP gene linked to higher levels of this protein have not been connected to a higher risk of VTE. This suggests that CRP displays the underlying inflammation, having no causal relationship with VTE [46]. Other studies have evaluated the diagnostic usefulness of CRP in VTE, with contradictory results: a prospective study suggested that CPR levels could be useful as a diagnostic biomarker in patients with suspected [47]; however, other prospective study showed that CRP combined with D-dimer did not improve the rate of negative imaging studies [48].

Six studies [53,54,55,56,57,58] have assessed the prognostic role of CRP in patients with PE. All of them were prospective and five of them measured CRP at diagnosis; however, the population size was highly heterogeneous, ranging from 35 to 276 patients, they pursued different objectives (mortality, VTE recurrence, or right ventricular (RV) dysfunction), and the follow-up periods were quite variable, ranging from 1 to 36 months. Only one study evaluated the short-term complications. In the first of these studies, Marchena-Yglesias et al. [53] measured CRP and other inflammatory biomarkers at VTE diagnosis in 100 patients, concluding that levels of CRP and other inflammatory markers might be useful to identify patients with a higher risk of mortality and complications during the first year of follow-up. In the second study, Chung et al. [54] did not find a correlation between CRP levels and platelet activation at diagnosis of VTE. On a similar note, Kline et al. [55] did not find a connection between CRP levels and mortality or RV dysfunction after six months of follow-up in 152 patients with hemodynamically stable PE. On the other hand, Abul et al. [56] noticed an association between CRP levels and RV dysfunction after three years, thus suggesting CRP might become a useful biomarker for risk stratification in PE. This study presented severe methodological limitations. In another study by Hogg et al. [57], CRP levels did not correlate to mortality in patients with VTE. Finally, in a recent prospective study by Demelo-Rodríguez et al. [58] on 586 patients with acute VTE (276 with acute PE), a cut-off point of 5 mg/dL for CRP on admission was associated with an increased risk for 30-day bleeding and mortality, in both the total sample and the subgroup of patients with PE, being independent of the simplified Pulmonary Embolism Severity Index (sPESI) and the European Society of Cardiology (ESC) score. This cut-off point improved their predictive capacity when combined with them.

Only two studies have evaluated the short-term prognostic role of CRP at diagnosis in patients with DVT. Gremmel et al. [59] evaluated the evolution of high-sensitivity CRP, plasmatic P-selectin, D-dimer, and Doppler ultrasound in 44 patients with acute unprovoked DVT, both at diagnosis and after 1, 3, 6, and 12 months of follow-up, compared with a cohort of 88 healthy individuals. At the moment of diagnosis, CRP and P-selectin were increased compared with the control population but significantly decreased after the first month. However, CRP remained similar to the control population after the first month, whereas both P-selectin and D-dimer could rise after the withdrawal of anticoagulation therapy, perhaps reflecting a prothrombotic state. The study by Demelo-Rodriguez et al. [58] included 358 patients with DVT. CRP levels measured at diagnosis were associated with 30-day mortality and 30-day bleeding risk in the total sample. A threshold level of 5 mg/dL for CRP was an independent risk factor for bleeding and mortality.

Three studies have evaluated the prognostic role of CRP in patients with cancer-associated thrombosis (CAT) but none on a short-term basis. The Vienna Cancer and Thrombosis Study (CATS) evaluated potential risk biomarkers for the development of VTE in cancer patients, showing that CRP was not an independent predictive factor for VTE, but it was a predictor of mortality [60]. Another study including 900 cancer patients found no association between CRP and the risk of VTE recurrence after the six-month follow-up. The three identified risk factors for VTE recurrence were TF levels, extrinsic venous compression, and hepatobiliary cancer [61]. Jara-Palomares et al. [62] assessed the role of CRP and high-sensitivity D-dimer after anticoagulation withdrawal in 114 patients with cancer-associated thrombosis. They concluded that both were potential biomarkers for the risk of VTE recurrence after anticoagulation discontinuation (HR: 9.8 (1.9–52) and 5.8 (1.1–31.7) for CPR and D-dimer, respectively). The previously mentioned study by Demelo-Rodríguez et al. [58] included 84 patients with CAT and showed that CRP levels were not associated with bleeding in this subgroup.

The relationship between CRP levels and prognosis in VTE patients could reflect an unspecific general severe condition [58]. This may be supported by the results of other studies demonstrating a similar prognostic role of CRP in acute infections [38], heart failure, or coronary disease [63]. In some cases, this rise might reflect a higher burden of morbidity and frailty rather than the severity of the acute disease [64]. On the other hand, a possible association between CRP levels at diagnosis of VTE and early complications seems reasonable, given the acute inflammatory mechanisms involved, rather than a relationship with long-term complications [53,54,55,56]. In summary, CRP is a promising biomarker for the stratification risk in patients with PE and more studies are necessary to validate the association between CRP levels and short-term mortality and bleeding.

### 3.2. P-Selectin

P-selectin is a cell adhesion molecule found on the surface of platelets and endothelial cells, that allows them to interact with leukocytes. P-selectin is also present dissolved in plasma [25].

In a case–control study [59] conducted in 44 patients with unprovoked DVT and 88 healthy controls, P-selectin levels were elevated at diagnosis and decreased after one month of anticoagulant therapy until its withdrawal. P-selectin levels rose again after stopping anticoagulation, suggesting a useful insight into the prevention of VTE recurrences.

Nevertheless, elevated levels of P-selectin at diagnosis of unprovoked VTE increase the risk of cancer diagnosis afterwards, with a high predictive capacity (C-statistic: 0.9 [0.83–0.97] for a threshold level of 45 ng/mL) [65]. Another study evaluated 43 potential biomarkers in patients with DVT. P-selectin showed the most distinct difference compared to healthy controls, and the authors suggested P-selectin levels might be related to the extension of thrombosis [66].

A prospective study assessing potential risk biomarkers for the development of VTE in cancer patients measured CRP and P-selectin in 705 patients with solid tumors with a follow-up of 12 months. The multivariate analysis demonstrated that only P-selectin remained as a predictor of VTE [60].

A meta-analysis of 11 studies comprising 586 patients with VTE and 1843 controls evaluated the diagnostic role of P-selectin [67]. P-selectin levels were elevated in VTE patients, regardless of the presence or absence of cancer, with an OR 2.88 (1.98–4.19), and showed an elevated diagnostic precision, with a specificity of 0.73 (0.51–0.90) and a C-statistic of 0.74. This offers advantages in situations where D-dimer is not useful since its positive predictive value is sufficient [68]. In combination with the Wells score, P-selectin shows a specificity of 96% and a positive predictive value of 100% for a Wells score ≥2 and P-selectin levels ≥90 ng/mL [69]. A following study validated these results [70]. However, contradictory results have been published in a prospective study that evaluated the predictive role of P-selectin for the development of VTE in patients with hematological malignancies [71].

The prognostic role of P-selectin in VTE patients has been scarcely evaluated. According to Chung et al. [54], soluble P-selectin levels are elevated in patients with PE. The echocardiographic RV ejection area correlated inversely with soluble P-selectin and positively with platelet P-selectin, which could indicate a dispersion of P-selectin from activated platelets related to the extent of the RV dysfunction. Regarding complications, P-selectin levels have shown no differences between patients with and without post-thrombotic syndrome (PTS) 51 months after DVT [72] and were not associated with VTE recurrence during a six-month follow-up in 900 patients with cancer and acute VTE [60].

In summary, P-selectin presents a promising biomarker for the diagnosis of VTE, given its high specificity, although the optimal threshold has not yet been defined and is still under investigation [68] and, as a diagnostic tool, P-selectin would need to be readily available with a quick turnaround. Its short-term prognostic usefulness remains unclear in PE patients.

### 3.3. Tissue Factor (TF) and Microparticles (MPs)

TF is a key receptor in the initiation of the blood coagulation cascade, leading to thrombin generation [73,74]. It is normally separated from the systemic blood circulation by the vascular endothelium and is expressed after stimulation of monocytes and endothelial cells by cytokines, endotoxins [23], and CRP [24]. MP are vesicles with a procoagulant surface that can originate from platelets, erythrocytes, monocytes, leukocytes, and cancer cells [75].

Considering that there is a poor standardization of analytical methods for MP detection [76], there is a discrepancy between the results of different studies. Many of them, including a recent meta-analysis [77], linked elevated levels of MP with the future occurrence of thrombosis, whereas others did not demonstrate its role as a predictive biomarker [78] or showed heterogeneous results. An association with the occurrence of VTE in pancreatic cancer might be present, whereas in other cancer entities, this could not be confirmed [75,79].

In a cohort of 900 patients with CAT evaluating CRP, D-dimer, factor VIII, TF, and P-selectin as predictors of VTE recurrence at six months [61], only TF was associated with VTE recurrence, and TF levels >64 pg/mL showed a HR 3.4 (2.1–5.5) for VTE recurrence.

There are no studies evaluating the short-term role of TF-MP in acute VTE. Regardless, its expression may support the metastatic potential of tumor cells [80] or the risk of VTE recurrence in cancer-associated VTE [61]. Therefore, elevated TF-MP levels may be associated with a worse prognosis in CAT patients.

### 3.4. Inflammatory Cytokines

Several studies emphasize the role of inflammatory markers such as interleukin (IL)-1, 6, 8, and 10 in VTE. By influencing the expression of TF, inflammatory cytokines provide a trigger that may lead to thrombotic disease [78]. Genetic association studies have reported possible links between inflammation-related genetic variants, especially cytokines (e.g., IL-1, IL-4, IL-10) [81] and VTE, thus establishing a role of genetic background in predisposition to VTE and variable inflammatory processes in individuals. IL-6 increases TF production and factor VIII transcription, along with fibrinogen production [82]. IL-8 induces TF production and adhesion of monocytes to the endothelium, thus inducing a procoagulant surface [83].

IL-6 levels have been reported to increase at the time of DVT diagnosis, without any correlation to the extent of the thrombosis, predisposing factors, or onset of symptoms [79]. Other authors have not found any association between IL-6 levels or genetic variants and VTE [84].

However, IL-10 has been described as a protective factor for VTE [85], and mutations in the IL-10 allele have been reported as an independent risk factor for VTE and recurrent VTE [78,86]. Elmoamly et al. [71] investigated TNF-α and IL-6, among other biomarkers. None of them were statistically associated with VTE in patients with hematological malignancies.

Few studies have evaluated the prognostic role of IL at diagnosis, with heterogeneous results. Marchena-Yglesias et al. [53] described an association between IL-6 levels and higher mortality in patients with VTE. Reitter et al. [87] found no correlation between VTE and IL levels in patients with newly diagnosed cancer or disease progression, but higher IL-6, IL-8, and IL-11 levels were associated with a reduced survival time in cancer patients.

Elevated IL-6 levels can independently predict the recurrence rate following DVT (OR: 1.66; 95% CI: 1.05–2.62). Moreover, increased levels of IL-6 following VTE are associated with an increased risk of VTE-related complications, in particular post-thrombotic syndrome (PTS), chronic thromboembolic pulmonary hypertension (CTEPH), and infections [88,89]. Van Aken et al. [90] detected elevated levels of IL-6 and IL-8 (OR: 2.4 and OR: 2.0, respectively) in patients with recurrent VTE. This was the first large clinical study showing that an increase in inflammatory mediators is associated with recurrent VTE.

In summary, few studies have evaluated the prognostic role of IL at VTE diagnosis, with heterogeneous results, and none of them assessed their short-term prognostic role specifically.

### 3.5. Erythrocyte Sedimentation Rate (ESR)

ESR has a role as an acute-phase reactant, although it rises more slowly and peaks later than CRP. It is also affected by other factors, including anemia, polycythemia, protein levels, red blood cell shape, patient age, or gender [91]. In a cohort of patients with hematological malignancies, an ESR level above 106 mm/h was the only biomarker that showed statistical significance as a predictor for VTE occurrence in patients with hematological malignancies [71]. Marchena-Yglesias et al. [53] found that a high ESR at the moment of VTE diagnosis protected against PTS at 12 months. The evidence on the prognostic value of ESR is scarce. There are no studies evaluating its short-term prognostic role in VTE.

### 3.6. Fibrinogen

Fibrinogen transforms to fibrin when TF induces thrombus formation, activating platelets. Some authors report no correlation between fibrinogen levels and risk of VTE, either in patients with or without hematological malignancies [44,71]. Others state that fibrinogen levels were indeed higher in patients with VTE than those without VTE [92]. However, fibrinogen levels were described as significantly higher in patients with unprovoked VTE than in those with risk-associated VTE and controls [93]. Lin et al. [94] propose that an age-adjusted approach for fibrinogen levels might be useful, in combination with other biomarkers, to detect surgical patients at risk of VTE development. As with other biomarkers, the prognostic role of fibrinogen in VTE patients has not been evaluated.

### 3.7. Leukocytes

White blood cells are involved in the inflammatory response of the organism. However, they are also involved in VTE development since they can keep the thrombi attached to the vessel wall [15], and leukocyte-derived particles were found to be related to increased thrombus formation [92]. An elevated leukocyte count has been suggested as an independent risk factor for in-hospital VTE despite thromboprophylaxis [95]. Blix et al. also stated that leukocytes may be directly involved in cancer-associated VTE [96].

When considering specific white cell populations, hypereosinophilia has been described as a potential risk factor for VTE [97], and monocyte-related mechanisms may be involved in VTE formation [98]. Elevated neutrophil counts have also been associated with an increased risk of VTE [99]. It is, however, the neutrophil-to-lymphocyte ratio (NLR) that has attained the most extensive research. Various authors have suggested that the NLR could be a useful predictor of VTE, whereas others have not found any association between NRL and VTE. Research suggests, additionally, that NLR may predict poor response to anticoagulation in patients with lung or gastric cancer and VTE [100]. As with other biomarkers, the prognostic role of leukocytes in VTE patients has not been evaluated.

**Table 2:** Clinical usefulness of inflammatory biomarkers.

**C-reactive protein**	↑ mortality and bleeding risk during the first month in VTE [58]; Improves predictive capacity of ESC and sPESI scores in PE [58]; ↑ mortality risk during the first 6–12 months (contradictory results) [53,55]; ↑ risk of RV dysfunction after 3 years in PE [56]; ↑ risk of PTS after 6 months (contradictory results) [53,92]; ↑ risk of VTE recurrence in cancer patients after discontinuation of anticoagulation [62]; Not associated with mortality in CAT [58].	
**P-selectin**	Elevated at VTE diagnosis (PPV 100% for a Wells score ≥ 2 and P-selectin levels ≥90 ng/mL) [68,69,70]; ↑ risk of TVE in cancer patients (contradictory results) [60,71]; ↑ risk of cancer in unprovoked VTE [65]; ↑ risk of RV dysfunction [54]; Possible association: VTE recurrences [59] and the extension of thrombosis [66]; Not associated with VTE recurrence in CAT after 6 months [60];	No studies have evaluated the short-term prognosis in VTE
**Tissue Factor-Microparticle**	Poor standardization of analytical methods for MP detection [76]; ↑ risk of VTE (contradictory results) [77,78]; ↑ risk of VTE recurrence in CAT after 6 months [61]; ↑ risk of metastatic cancer in oncologic patients [80].	
**Inflammatory cytokines**	IL-6: Not associated with ↑ risk of VTE in oncologic patients; IL-6: ↑ risk of mortality during the first year after VTE [53]; IL-6: ↑ risk of DVT recurrence, PTS and CTEPH [88,89,90]; IL-8: ↑ risk of VTE recurrence [90]; IL-10: protective factor for VTE [85]; Lower levels of IL-10: ↑ risk of VTE and VTE recurrence [78,86].	
**Erythrocyte sedimentation rate**	Levels >106 mh: ↑ risk of VTE in hematological malignancies [71]; ↓ risk of PTS 1 year after a DVT (probably biased) [53].	
**Fibrinogen**	↑ levels in unprovoked VTE compared with risk-associated VTE [93].	
**Leukocyte count**	↑ risk of in-hospital VTE despite thromboprophylaxis [95]; Hypereosinophilia: ↑ risk of VTE [97]; Neutrophil to lymphocyte ratio: ↑ risk of VTE (contradictory) [99,100].	

CAT: cancer-associated thrombosis; CTEPH: chronic thromboembolic pulmonary hypertension; DVT: deep venous thrombosis; ESC: European Society of Cardiology; MP: microparticles; PE: pulmonary embolism; PPV: positive predictive value; PTS: post-thrombotic syndrome; RV: right ventricle; sPESI: simplified Pulmonary Embolism Severity Index; VTE: venous thromboembolism.

## 4. Prognostic Biomarkers for Bleeding Risk during Anticoagulation in VTE

The most relevant adverse effect of anticoagulation therapy is bleeding, which may be fatal. The highest risk occurs during the first seven days of treatment. Major bleeding in PE occurs in 3–4% of the cases, whereas in DVT it occurs in barely 0.1% [101]. However, the bleeding risk depends on the anticoagulant therapy of choice: direct oral anticoagulants, compared to antivitamin K, show a lower risk of major, fatal, intracranial, and clinically relevant nonmajor bleeding, along with overall bleeding risk, and they do not increase the risk of digestive bleeding.

Some risk factors have (gender, age, cancer, recent major bleeding, etc.) been proposed and used in different clinical–analytical scores to stratify bleeding risk in patients with acute VTE (Kuijer score, RIETE 3-month, RIETE 10-day, VTE-BLEED, and ACCP clinical guideline scores), with heterogeneous and contradictory results, lacking sufficient evidence and validation. None of them could determine the risk of major bleeding in patients with VTE under anticoagulation therapy. Most of these scores include clinical variables, but only a few include analytical parameters. Only a few laboratory markers have shown to be associated with a higher bleeding risk in VTE patients: altered prothrombin time, thrombocytopenia, anemia, creatinine clearance levels <30 mL/min, and D-dimer [102,103,104].

Among inflammatory biomarkers, only CRP has been evaluated as a bleeding risk factor in patients with VTE. In the study by Demelo-Rodríguez et al. [58], CRP levels were associated with 30-day bleeding, and C-statistic was 0.64 (CI 95%: 0.60–0.68). Marchena-Yglesias et al. [53] found no association between CRP levels and bleeding at 12 months of follow-up.

In conclusion, there is scarce evidence regarding the predictive capacity of inflammatory biomarkers for bleeding in VTE patients. Specifically, there are no data on patients with cancer-associated thrombosis.

## 5. Conclusions

In the past 15 years, only a few studies evaluating the prognostic role of inflammatory biomarkers in patients with VTE have been published, with indefinite results. Nonetheless, some of them suggest that the elevation of certain biomarkers, transient or maintained, both at diagnosis or during follow-up, may be useful to identify patients at risk of early complications (mortality, recurrence, and bleeding), especially in acute PE. However, most of them are involved in different mechanisms, which reduces their specificity. Only CRP has demonstrated an association with short-term mortality and bleeding, although these results need to be externally validated. Moreover, some of the reported inflammatory biomarkers are not widely available in clinical practice. Of them, P-selectin is the most promising one, although it is not widely available. Some authors have reported its potential usefulness in VTE, with high specificity, especially in cancer patients. However, its prognostic capacity has not yet been defined and warrants further research.

## Figures and Tables

**Table 1:** The role of different inflammatory biomarkers in the pathophysiology of venous thromboembolism (VTE).

Biomarkers	Role in the Pathophysiology of VTE
**C-reactive protein**	Its production is stimulated by cytokines. It modulates innate immunity.
**P-selectin**	Cell adhesion molecule present in platelets and endothelial cells. It mediates the binding of platelets and endothelial cells with leukocytes and the transfer of TF to platelets and triggers the formation of leukocyte-derived microparticles.
**Microparticles**	Small membranous vesicles, released from the plasma membranes of platelets, leukocytes, red cells, and endothelial cells. They play an important role in the initiation and propagation of VTE through the development of their own procoagulant properties, enhancing intercellular communication and promoting inflammation.
**Interleukins**	Released by leukocytes, endothelial cells, and other cell types that promote inflammation, they influence endothelial function and the expression of TF.
**Fibrinogen**	Induces thrombus formation through platelet activation.
**Leukocytes**	They can keep the thrombi attached to the vessel wall. Leukocyte-derived particles were found to be related to increased thrombus formation.

TF: tissue factor; VTE: venous thromboembolism. Adapted from: Anghel L, Sascău R, Radu R, Stătescu C. From Classical Laboratory Parameters to Novel Biomarkers for the Diagnosis of Venous Thrombosis. Int J Mol Sci. 2020;21(6):1920.

## Data Availability

The study did not report any original data.
